# Cord Lining Mesenchymal Stem Cells Have a Modest Positive Effect on Angiogenesis in Hindlimb Ischemia

**DOI:** 10.3389/fcell.2020.596170

**Published:** 2021-03-08

**Authors:** Kenon Chua, Fui Ping Lim, Victor Kwan Min Lee, Toan Thang Phan, Bee Choo Tai, Yih Kai Tan

**Affiliations:** ^1^Programme in Cancer and Stem Cell Biology, Duke-NUS Medical School, Singapore, Singapore; ^2^Department of Surgery, Yong Loo Lin School of Medicine, National University of Singapore, Singapore, Singapore; ^3^Alice Lee Centre for Nursing Studies, National University of Singapore, Singapore, Singapore; ^4^Department of Pathology, National University Hospital, Singapore, Singapore; ^5^National University Cancer Institute, Singapore, Singapore; ^6^Department of Pathology, Yong Loo Lin School of Medicine, National University of Singapore, Singapore, Singapore; ^7^Saw Swee Hock School of Public Health, National University of Singapore, Singapore, Singapore; ^8^Novena Vascular and Varicose Vein Centre, Mount Elizabeth Novena Specialist Centre, Singapore, Singapore; ^9^Department of Surgery (Vascular), Changi General Hospital, Singapore, Singapore

**Keywords:** cell therapy, stem cell, mesenchymal stem cell, critical limb ischemia, peripheral arterial disease, hindlimb ischemia, angiogenesis

## Abstract

**Purpose:** We investigated the use of human Cord Lining Mesenchymal Stem Cells (CL-MSCs) (US Patent number 9,737,568), in a rabbit hindlimb ischemia model, and evaluated their potential in stimulating neovascularization. Allogenic human CL- MSCs could potentially be used to treat patients with lower limb ischemia and non-healing wounds.

**Methods:** Twenty rabbits were divided into two separate groups. We created a hindlimb ischemia model surgically. At 21 and 49 days post-operatively, animals in the treatment group were injected with CL-MSCs (500,000 cells per 0.2 ml on each site) at 10 different sites (Quadriceps- 4 sites, Hamstrings- 4 sites and Calf-−2 sites) in the hindlimb muscles. The control group received only saline injection to the corresponding sites at the same time point as the treatment group. We then evaluated the effects of treatment on neovascularization by angiography, laser doppler perfusion imaging, as well as by histology. We evaluated the tissue samples for any signs of local immune reaction to the cell implantation. We also observed the rabbit clinically for any adverse effects after treatment.

**Results:** We found a higher number of CD31 positive cells in the treatment group, with a greater number of capillaries found in the treated muscles. The Rectus Femoris demonstrated a median vessel count/muscle fiber of 0.121 for the treatment group, compared to 0.076 in the control group (median difference 0.04; 95% CI 0.001–0.11; *p* = 0.041). The Gastrocnemius demonstrated a median vessel count/muscle fiber of 0.175 for the treatment group, compared to 0.089 in the control group (median difference 0.087; 95% CI −0.006 to 0.234; *p* = 0.07). Blood perfusion quantification through Laser Doppler Perfusion Imaging (LDPI) also demonstrated a non-statistically significant increase in perfusion in favor of the treatment group. CL-MSCs demonstrated no toxicity associated morbidity and minimal local immune reaction to implantation.

**Conclusion:** CL-MSCs have a positive effect on angiogenesis in a rabbit hindlimb ischemia model. This preliminary data is encouraging and paves the way for future large animal studies or for clinical trials.

## Introduction

Critical limb ischemia (CLI) is regarded as one of the most detrimental forms of peripheral artery disease with high rates of disability and mortality (Tu et al., 2015; Shishehbor et al., 2016; Teraa et al., 2016). Globally, CLI has affected more than 200 million people worldwide, with lower-middle income countries accounting for an increase in prevalence rate of 29%, and higher income countries reflecting a 13% increase (Fowkes et al., 2013; Sampson et al., 2014; Jelani et al., 2018). Significantly, this was closely associated with a 1-year major amputation rate of 40% (Ryu et al., 2012; Ponemone et al., 2017), while mortality rates increased by 20% within 6 months of diagnosis and 50% after 5 years of diagnosis (Norgren et al., 2007; Teraa et al., 2016). The current standard therapy aims to increase blood circulation to the affected limb either through surgery or endovascular revascularization (Tretinyak et al., 2001; Adam and Bradbury, 2007). However, an estimation of 20–50% of patients are high-risk surgical patients or with undesirable endovascular anatomy which limit their current interventional options (Dormandy et al., 1999; Idei et al., 2011). In addition, postoperative arterial re-occlusion is a rapid occurrence which further limits the intervention, and leaves CLI patients with no ideal alternatives for intervention (Lawall et al., 2011).

While pharmacological interventions such as antilipidemic, antiplatelet and antihypertensive therapies have been used to address the underlying state of atherosclerosis, none of these interventions have resulted in a decrease in amputation rates in patients with CLI (Conte et al., 2009; Gupta et al., 2014). Presently, the US Food and Drug Administration (FDA) has no authorized treatment for CLI. Often, the only choice that remains for no-option CLI patients will be treatment directed toward pain management, wound care, and eventually limb amputation. Taking into account the limiting circumstances of present interventions and its soaring mortality figure, the quality of life for CLI patients is not dissimilar to that of terminal cancer patients (Powell et al., 2011). These patients are left with no treatment options and constitute a population of patients with a detrimental and potentially fatal condition, as well as an unfulfilled medical need. Clearly, there is a pressing call to make headway for an alternative therapeutic approach to treat this intractable disease (Liew and O'Brien, 2012).

In the last two decades, there has been an exponential increase in the number of articles detailing the potential effects of stem cell therapy and featuring it as an up-and-coming alternate treatment for CLI (Lawall et al., 2010; Sprengers et al., 2010; Powell et al., 2011; Schiavetta et al., 2012; Wang et al., 2014). It has been reported in several studies that Mesenchymal Stem Cells (MSCs) express multiple proangiogenic growth factors; such as VEGF, VEGFR, ANG-1 and SDF-1, and can migrate to hypoxic areas, thereby enhancing angiogenesis and restoring the vasculature network in animal models of hindlimb ischemia (Liang et al., 2014, 2017). In clinical studies involving human volunteers, the administration of bone marrow cells has yielded favorable outcomes in patients with CLI; such outcomes include an improved Arterial Brachial Index and Transcutaneous Oxygen Pressure, an improved limb perfusion, increased walking distance, reduced pain and ultimately, reduced rate of major amputation, improved overall ischemic symptoms, and quality of life (Tateishi-Yuyama et al., 2002; Kajiguchi et al., 2007; Lawall et al., 2011; Powell et al., 2011). A recent systematic review that appraised several studies which had used cell-based therapy on patients with CLI reiterates a similar impression that cell-based treatment is effective in reducing amputation rate, improving perfusion, and prolonging the amputation-free survival (Rigato et al., 2017).

MSCs have been successfully harvested from several tissue types, namely, the bone marrow (BM), adipose tissue (AT), umbilical cord (UC) tissue, and umbilical cord blood (UCB) (Kern et al., 2006; Kim et al., 2007; Cho et al., 2009; Lv et al., 2012; Luo et al., 2017). However, the clinical adoption of BM, AT, UC, and UCB—derived MSCs is limited by certain drawbacks. Aged cells from older patients are less biologically active and have a limited capacity for proliferation and differentiation. Cell extraction involves invasive procedures with significant donor site morbidity (Chamberlain et al., 2007; Dimmeler and Leri, 2008). A significant delay is also involved during cell culture and expansion. To overcome the pre-existing difficulties in the translational use of stem cells, we will be using a novel source of MSCs derived from human cord lining (US Patent number 9,737,568). In our previous study, we demonstrated that these Cord Lining Mesenchymal Stem Cells (CL-MSCs), when compared with MSCs derived from other gestational tissues; namely umbilical cord blood (CB-MSCs), placenta (P-MSCs), and Wharton's jelly (WJ-MSCs); have showed the highest proliferation and migration rate as well as prolongation in survival, which is attributed to their ability to dampen TH1 and TH2 responses (Stubbendorff et al., 2013). Moreover, CL-MSCs, when compared to other gestational tissues, yielded the lowest immunogenicity, which is correlated with lower HLA 1 expression (Stubbendorff et al., 2013), as well as its ability to reduce the release of IFN-gamma in mixed lymphocyte reaction (Stubbendorff et al., 2013). Similarly, CL-MSCs when compared with bone marrow MSCs (bmMSCs) have significant lower HLA 1 expression, higher production of tolerogenic TGH-beta and IL-10, and highest proliferation rate (Deuse et al., 2011). Altogether, the CL-MSCs are superior cells, with the most promising potential for cell based therapy because of their higher proliferative capacity, lower immunogeneity, and stronger immunosuppressive potential. This cGMP grade CL-MSCs or CorLiCyte has been approved by US-FDA for Phase-1 clinical trial for non-healing diabetic wounds and is believed to be a promising alternative source of stem cells. It offers a practical and affordable source of cells for cell-therapy for peripheral vascular disease. Besides the biological differences compared to other MSCs, they are processed from a rich source of cells (umbilical cord lining) which is routinely discarded after birth. This reduces the economic cost and logistic burden of therapy, facilitating greater adoption, and ability to penetrate into populations where financial resources may be scarce. This can potentially lower the incidence of major amputation in CLI patients.

To the best of our knowledge, the functionality of this novel MSCs on ischemic hindlimb has not been evaluated. Our study represents the first investigation of the therapeutic effect of CL-MSCs on hindlimb in a rabbit model. We investigated the use of CL- MSCs in hindlimb ischemia in a rabbit model and evaluated their potential in generating neovascularization. We predict that the rabbits in the treatment group (standard care with CL-MSCs) will demonstrate evidence of increased neovascularization compared to the control group (standard care with no CL-MSCs). We also predict that the rabbits in the treatment group will demonstrate evidence of increase functional collateral network formation compared to the control group.

## Materials and Methods

### Cord Lining Mesenchymal Stem Cells Isolation, Characterization and Culture

CL-MSCs isolation and cultivation were performed according to the patented protocol by CellResearch Corp Pte. Ltd. (US Patent number 9,737,568). Stem cell characterization and quality control were performed in compliance with cGMP processing protocol that has been submitted to US-FDA and is approved for clinical trials on human subjects.

Briefly, CL-MSCs were isolated from the outer amniotic lining of the umbilical cord and were cultured in PTT4 media (CellResearch). Cells were grown in their specific growth media [CMRL-1066 + antibiotics + L-glutamine] (Cell Research Corp). Cells were split at a confluency of 80–85% by mechanically lifting the cells from the tissue culture plastic surface using a cell lifter (Costar, Corning). Before transplantation, MSCs were dissociated by collagenous type 1 solution at a concentration of 0.025% for 5 min at 37°, and resuspended in sterile PBS at 1 × 10^6^ cells per 50 μl. MSC viability was ~95% as determined by trypan blue staining.

### Preparation of Rabbit Hindlimb Ischemia Model

**Figure 1:**
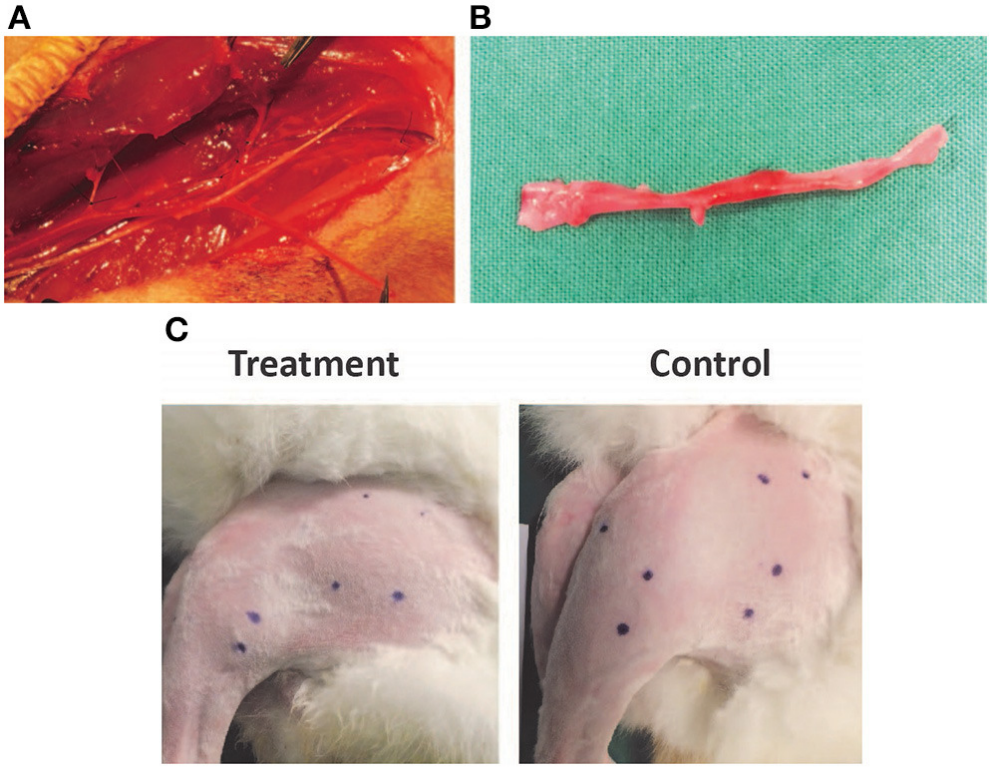
**(A,B)** Dissection and ligation of the superficial femoral artery in the rabbit hindlimb. **(C)** Injection sites in the rabbit hindlimb muscle bellies marked prior to treatment to ensure consistent localization.

This study was approved by the Institutional Review Board and SingHealth Institutional Animal Care and Use Committee (#: 2013/SHS/ 836). Twenty male rabbits (New Zealand White strain) of weight 2.5–3 kg, ~11–13 months in age, were used in this experiment. Hindlimb (left) ischemia in the rabbits was created by surgical ligation of the common femoral artery (CFA). The right hindlimb was kept intact. A longitudinal skin incision on the medial aspect of thigh, from the inguinal ligament to the knee, was made in the left hindlimb. The CFA, superficial femoral artery (SFA), and profunda femoral artery were dissected, ligated (with 6/0 prolene) and divided. SFA was then dissected down to popliteal and saphenous arteries. Popliteal and saphenous arteries as well as their associated branches were ligated (with 6/0 prolene) and divided at the knee level. The entire length of SFA and part of the popliteal artery and the saphenous artery were excised and discarded (Figures 1A,B).

Twenty rabbits were divided into two separate groups. Group 1 was the treatment arm. At 21 and 49 days post-operatively (after the establishment of hindlimb ischemia model), animals in Group 1 were injected with CL-MSCs (500,000 cells per 0.2 ml on each site) at 10 different sites (Figure 1C; Quadriceps- 4 sites, Hamstrings- 4 sites and Calf-−2 sites) of the ischemic left hindlimb muscles. Group 2 was the control group which received only saline injection to the corresponding sites in the ischemic left hindlimb muscles, at the same time point as group 1. The effect of CL-MSCs compared to control was monitored using laser doppler flowmeter (Imager) over 15 weeks (at week 7, 11, and 15) following the first treatment at week 3. The presence of constitutional symptoms such as fever, loss of appetite, loss of weight, movement, and rabbit grimace scale were documented throughout the experiment. At week 15 post-operation, all the animals were euthanized. Peripheral angiography was performed at this time point. The left hindlimb Rectus Femoris muscle and Gastrocnemius muscle were harvested for histology analysis.

### Angiography

Peripheral angiography by C-Arm machine was performed before animal euthanasia in a lead-lined section of the experimental operating theater. Each rabbit was anesthetized. Midline laparotomy was performed and small bowel was mobilized to expose the abdominal aorta. The aorta was cannulated with a 4-French Micro sheath (Angiodynamics) using the Seldinger technique. Digital Subtraction Angiography was carried out with 2.5 ml of contrast (Visipaque) being injected via the 4-French catheter. The distance between the image intensifier and the table was fixed at 25 cm for the angiography performed. Region of Interest (ROI) was drawn in the area of the medial thigh to include the newly developed collaterals. Same ROIs were used in all limbs in order to reproduce equal sampling areas. Quantification of neovascularization was done by standard grid overlay counting of collateral vessels in the region of interest. Analysis was done by 1 independent operator blinded to the treatment.

### Laser Doppler Perfusion Imaging

The total local microcirculatory blood perfusion is measured using Perimed Laser Doppler Perfusion Imaging (LDPI) at week 7, 11, and 15 post-surgical ligation of the CFA. Each animal is sedated using Ketamine (50 mg/kg) and Xylazine (10 mg/kg) intramuscularly prior to the imaging procedure and maintained under inhalational anesthesia [Isoflurane (1–2%)] during the procedure. The hair from the hindlimb is removed using an electric shaver followed by hair removal cream as necessary. The measurements were expressed as perfusion units (PU).

### Histology

The muscle tissues were dissected from the hindlimbs of euthanized rabbits, and consecutively fixed in formalin. Two muscle groups, the Rectus Femoris and Gastrocnemius, were analyzed using histopathologic procedure. Specimens were embedded in paraffin and sectioned. To locate and to mark up the endothelial cells and capillaries, the IHC staining for CD31 using UltraView DAB polymer kit (Dako-Immunoglobulins ApS, Produktionsvej, 42, 2600, Glostrup, Hovedstaden Denmark; Dilution of 1/100) was performed on Roche Ventana Ultra Automated machine (Retrieval: CC1-64 min; Incubation time: 32 min; Incubation temp: 37°C). The tissues were further stained with CD68 antibody (Boster Biological Technology Co., Ltd., Wuhan, China; Dilution of 0.4 ug/ml; Incubation time: 30 min; Incubation temp: 36.9°C) and evaluated by a clinical pathologist to look for evidence of inflammation and fibrosis. All samples were coded. Unbiased histological examination was performed by a pathologist, blinded to the treatment group. Five microscopic fields (400X magnification) with increased capillary density were randomly selected and the number of stained capillaries and fibers within each field were quantified using manual counting. The values were represented using median number of vessel count/muscle fiber (V/M) from two sections 200 μm apart.

### Statistical Analysis

The difference in median (V/M) ratio between groups was compared using the Wilcoxon rank sum test. The effect of treatment on perfusion over time was evaluated using the linear mixed model with random intercept, to take into account possible intra-subject correlation in the serial measurements. Natural log transformation was carried out to normalize the perfusion data. All statistical analyses were generated using STATA V14, assuming a two-sided test at the 5% level of significance.

## Results

### Cord Lining MSCs Injections Had No Increased Morbidity or Local Reaction

**Figure 2:**
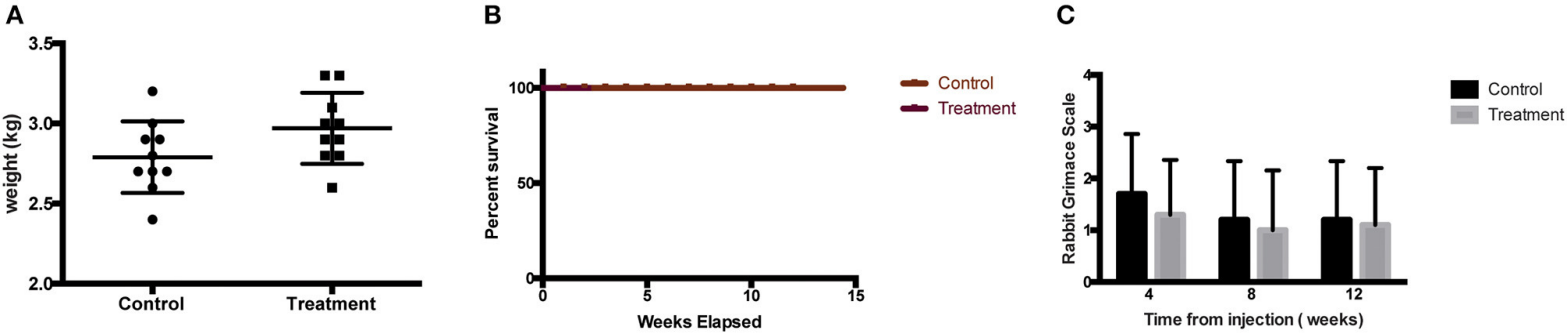
**(A)** Weight of rabbit at final time point before sacrifice demonstrated no difference between control and treatment groups. **(B)** Kaplan Meier survival graph for treated rabbit demonstrated no difference between control and treatment groups. **(C)** Rabbit Grimace Scale demonstrated no difference between control and treatment groups.

**Figure 3:**
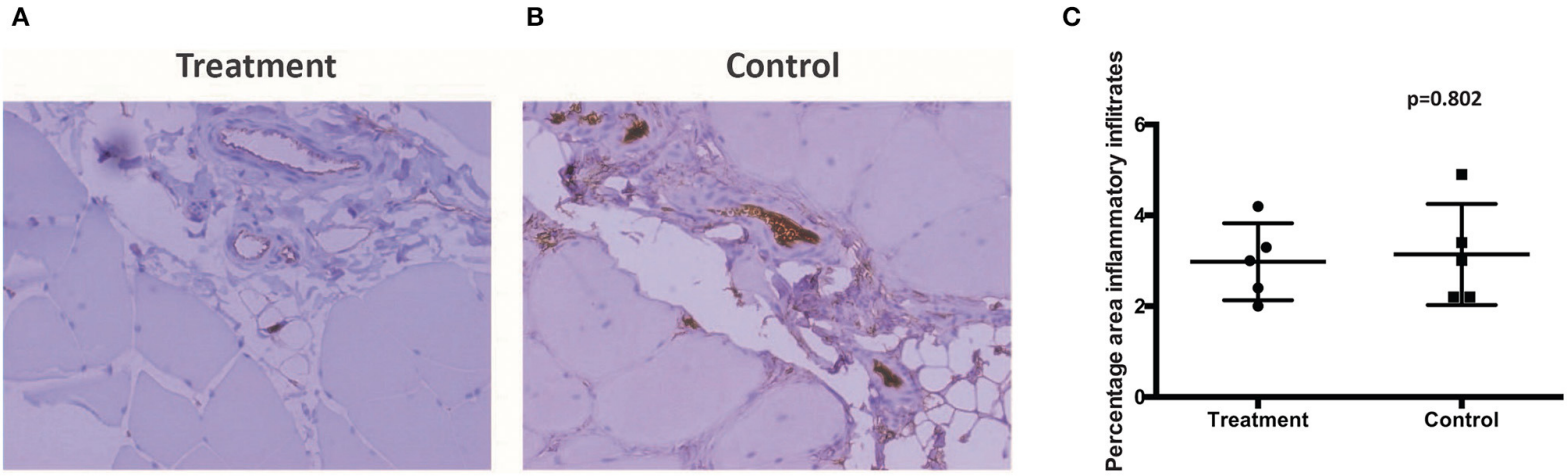
**(A,B)** Muscle samples taken at the injection site demonstrated no difference in percentage inflammatory infiltrate, fibrosis, or local reaction between control and treatment. **(C)** Proportion of infiltrated inflammatory cells between control and treatment.

There was no report of adverse or serious condition occurring in both groups of rabbits. All surgical wounds healed with no complications and there were no soft tissue tumors noted at the injection site for both the treatment and control groups. The rabbits in the treatment arm experienced no adverse changes in temperature or mobility. There is no difference in the weight of rabbits between the control and the treatment groups (Figure 2A). Animals in the treatment arm had a 100% survival rate to the end of the experiment (Figure 2B). The rabbit grimace scores suggested minimal post implantation pain and were also comparable for both groups (Figure 2C). Macroscopic evaluation on the sites of CL-MSCs injection showed no tissue necrosis or foreign body reaction. There was a minimal increase (<5%) in inflammatory cells or fibrosis between the two groups (Figures 3A–C). This indicates no gross immune response on the injected tissue.

### Cord Lining MSCs Injections Resulted in Increased Angiogenesis in the Rectus Femoris in the Ischemic Limb

Gross microscopic evaluation of the Rectus Femoris muscle and Gastrocnemius muscle from both the control and treatment groups showed no significant ischemic or fibrous scarring after ligation of the SFA and at termination of the experiment. This indicates that the rate of angiogenesis and existent collateral blood flow is adequate to prevent tissue necrosis after ligation, even in the non-treatment arm in our hindlimb ischemia model. As expected, both groups demonstrated no inhibition in mobility after ligation secondary to lower limb muscle necrosis.

**Figure 4:**
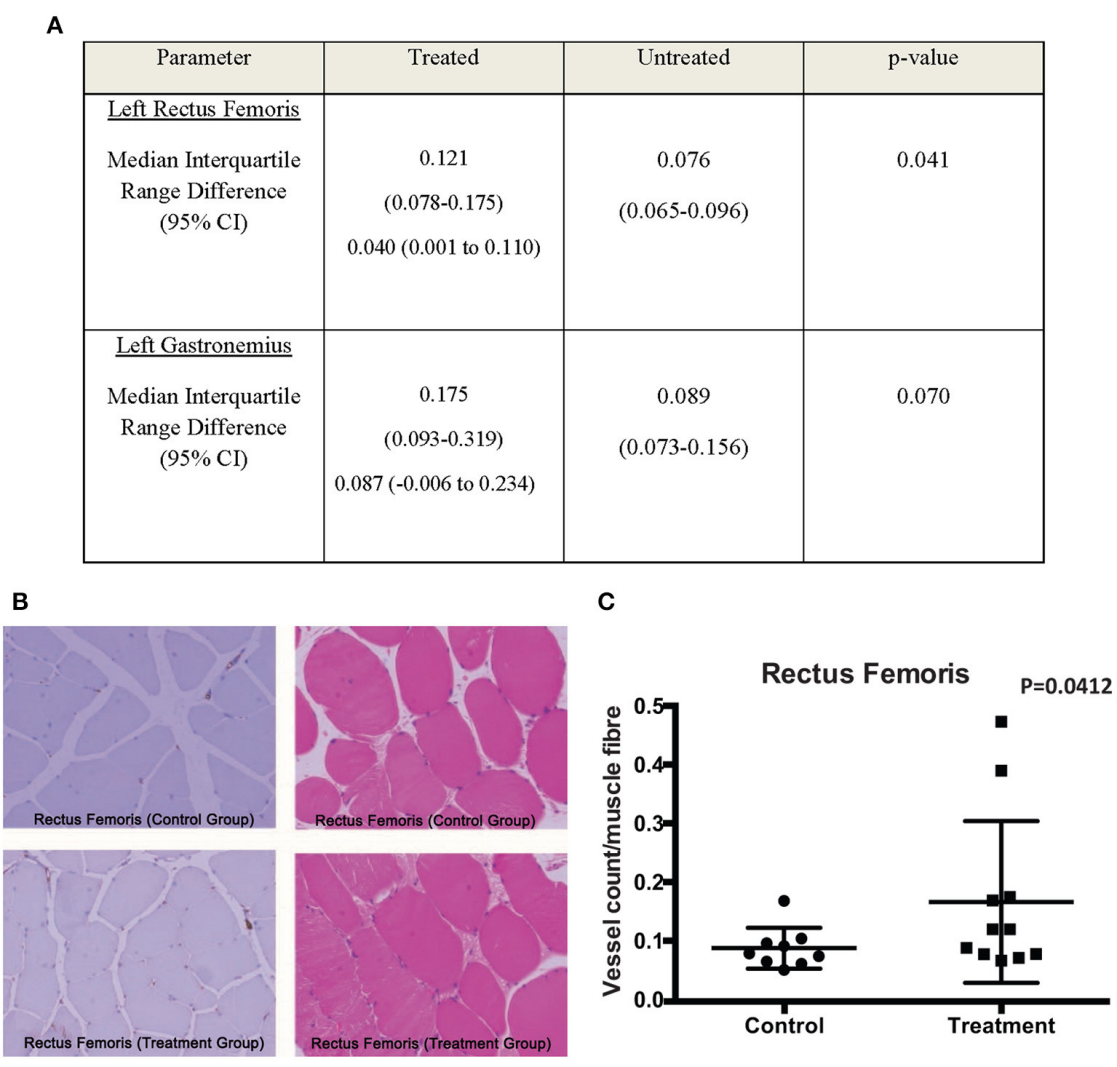
**(A)** Comparison of median V/M ratio between treated and untreated groups. **(B,C)** CD31 and H&E stains of the Rectus Femoris muscles demonstrated a trend toward increased V/M ratio scores for the treatment group compared to the control group.

Of interest is the increase in angiogenesis in the Rectus Femoris and Gastrocnemius in the treatment arm. The Rectus Femoris demonstrated a median vessel count/muscle fiber of 0.121 in the treatment group, compared to 0.076 for control group (median difference 0.04; 95% CI 0.001–0.11; *p* = 0.041; Figures 4A–C). The Gastrocnemius demonstrated a median vessel count/muscle fiber of 0.175 in the treatment group, compared to 0.089 for control group (median difference 0.087; 95% CI −0.006 to 0.234; *p* = 0.07; Figures 4A–C). The difference in Rectus Femoris but not the Gastrocnemius is significant as it indicates that the CL-MSCs injections had an effect on the rabbit angiogenesis after ligation of the SFA in the proximal muscles but not the distal muscles. This is not surprising, as the majority of the injections for implantations were done in the proximal muscles (8 injection sites) compared to the distal muscles (2 injection sites). This suggests that the therapeutic effect of the CL-MSCs on angiogenesis in ischemic muscles is likely to be dependent on cell quantity.

### Cord Lining MSCs Injections Resulted in Marginal Increase in Perfusion in Hindlimb Ischemic Limbs

**Figure 5:**
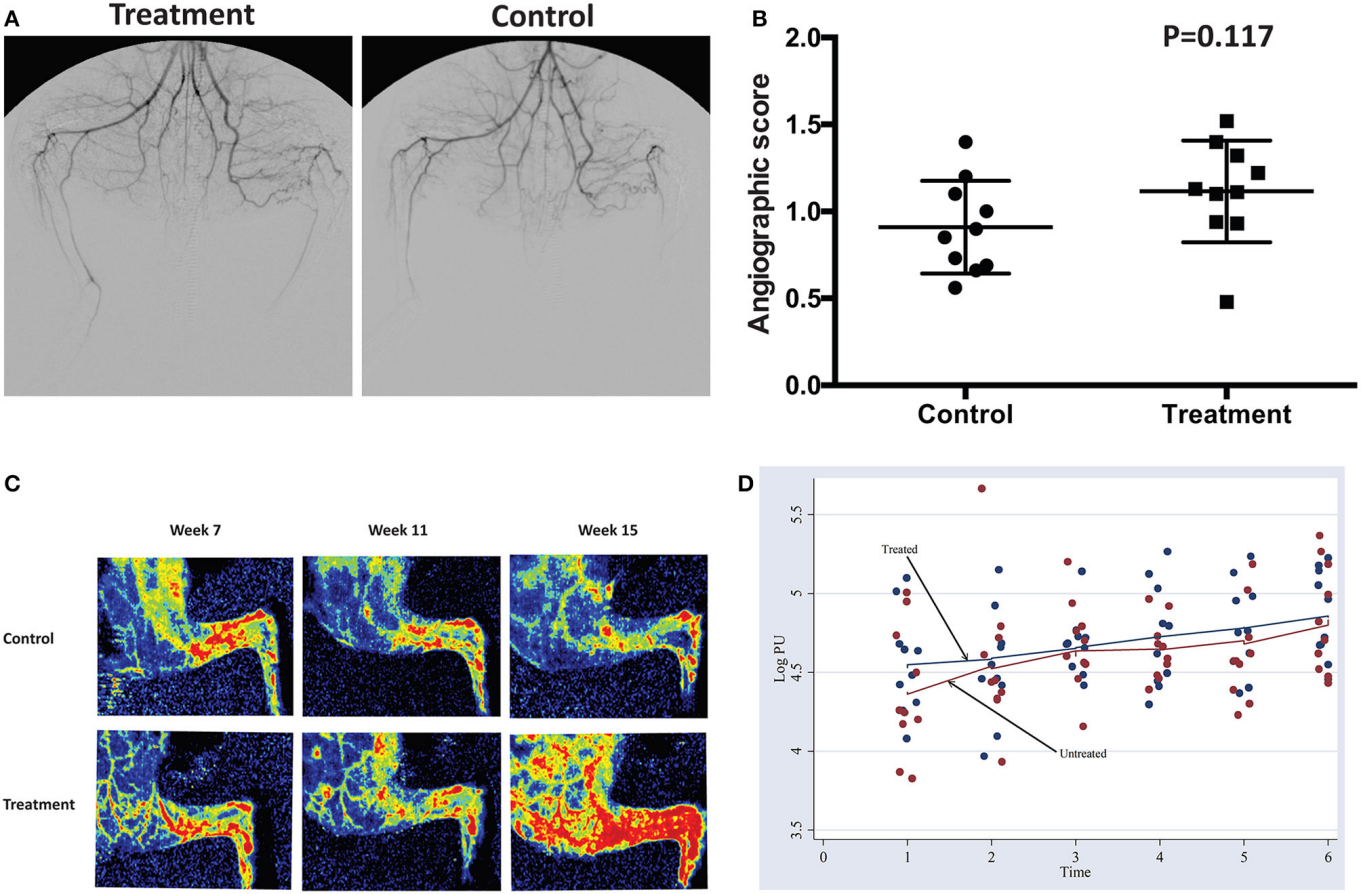
**(A,B)** Peripheral angiography performed at week 15 before euthanasia demonstrated a weak trend toward a higher angiographic score in the treatment compared to the control group. **(C)** Laser Doppler perfusion Imaging of the hindlimb at week 7, week 11 and week 15 demonstrated a trend toward progressively more flow in the treatment group. **(D)** Pattern of change in logPU over time.

Peripheral angiography revealed a marginal increase in perfusion toward the treatment group. However, it did not demonstrate a statistically significant difference between groups (Figures 5A,B). Similarly, images acquired through LDPI revealed a slight increase in perfusion toward the treatment group (Figure 5C). There is no significant difference in the Log Perfusion Unit (PU) between treated and untreated groups. Both groups demonstrate an uptrend pattern in the LogPU over time, with the treated group projecting a slightly steeper slope (Figure 5D).

## Discussion

In this observational report, we have demonstrated that CL-MSCs have low morbidity associated with systemic toxicity. In our histology analysis, we have established that injection of CL-MSCs did not trigger adverse tissue response. This is an important first step in establishing the safety profile of this treatment modality in preparation for pre-clinical trials. Any cell-based therapy has the potential for immune rejection and inflammation. This is of even greater concern in the use of non-autologous cells, such as CL-MSCs. CL-MSCs have the practical advantage of being bankable from different donors in large numbers. This ensures that therapeutic cells are of low passage number. They are derived from the umbilical cord lining and hence have an inherently low immunogenicity.

We have also demonstrated that there is significant effect in the stimulation of angiogenesis in the hindlimb ischemia rabbit model in quantitative histological outcomes, which were the most sensitive and least subjective investigative technique we had. Due to the phase dependent nature of the clinical angiogram, we were not able to get a statistically significant result in this particular investigative modality. LDPI revealed a marginal increase in LogPU over weeks but did not demonstrate a significant difference between groups. LDPI is a crude measurement that is subject to multiple confounders. This includes varying degrees of fur covering the limb, environmental or animal core and peripheral temperature, as well as ambient light. It may not be adequately sensitive to determine small quantitative differences in perfusion but it does have the advantage of being non-invasive and repeatable over time. The fact that the Rectus Femoris (proximal) but not the Gastrocnemius muscle (distal) demonstrated a significant difference in the degree of angiogenesis between the treatment and control suggests that the cell therapy has a localized effect rather than a longer range or systemic effect. This is consistent with other reports on the mechanism of action of MSCs (Powell et al., 2011; Schiavetta et al., 2012; Liang et al., 2017). CL-MSCs should have a more consistent clinical efficacy compared to marrow or adipose derived stem cells as they are not taken from aging donor (peripheral vascular disease is typically a disease of the middle aged or older adults) and can be available in large numbers. There is also no waiting time for cell expansion in contrast to bone marrow derived cells which can take up 2–3 weeks for cell expansion. This gives CL-MSCs a huge advantage in the treatment of conditions that are more time sensitive and in avoiding a missed therapeutic window.

The characterizations of CL-MSCs were well-established and showed typical MSC characteristics in previous reports. Flow cytometry clearly showed that the isolated cells expressed CD44, CD73, CD90, and CD105 on the cell surface, confirming the qualifying criteria of MSCs (Kita et al., 2010; Deuse et al., 2011; Martinez et al., 2013; Stubbendorff et al., 2013). The prominent feature of CL-MSCs is that the cells also express several Stem Cells (SC) markers in addition to MSC markers, namely, noteworthy that 100% of the cells expressed Nanog, which is one of the key molecules necessary for the maintenance of self-renewal of SCs, and Oct-4, another well-accepted molecule to define SCs (Stubbendorff et al., 2013). Many of our cells expressed SSEA-4 (Kita et al., 2010; Deuse et al., 2011; Stubbendorff et al., 2013), which is shown to have superior propagative activity, consistent with the observation of the numerous formation of colonies (~30 colonies, each larger than 2 mm in diameter), again indicating the CL-MSCs retain the capacity to propagate and actively migrate during proliferation. Another general defining criteria of CL- MSCs is the ability to differentiate into at least 3 lineages; osteogenic, adipogenic and chondrogenic (Martinez et al., 2013; Stubbendorff et al., 2013). Further, CL-MSCs did not show anchorage-independent growth, indicating that CL-MSCs did not show any tumor cell-specific features, an important aspect for future applications of CL-MSC in regenerative medicine.

There were certain limitations in the scope of our study. This study did not investigate the mechanism of action of the CL- MSCs. The mechanism of action of MSCs in angiogenesis has been well-elucidated and characterized in many other studies (Lawall et al., 2010; Sprengers et al., 2010; Powell et al., 2011; Schiavetta et al., 2012; Liang et al., 2014, 2017). We also did not investigate serum and organ specific changes during treatment. This would have been important in ensuring that besides the local immune reaction, there is no minor systemic toxicity, which may not have been exhibited in morbidity or symptoms from the rabbits. However, the physiological outcome measurements documented in our study are adequate for us to demonstrate that there was no major local or systemic reaction to the cell therapy.

Another limitation is that the microenvironment of the hindlimb ischemia model may not be identical to the clinical presentation. Even in the control arm, all the subjects showed negligible ischemic changes in the muscle after vascular ligation. This may be due to extensive collateral flow, or the rapidity of neovascularization in rabbits. However, since tissue hypoxia is known to be important in cell signaling and angiogenesis, this difference may account for the blunted therapeutic response in our treatment arm, compared to the control arm. Lastly, we did not label the implanted cells as there were concerns over how cell labels might markedly change the nature of our CL-MSCs and confound our findings. Further, the survival of CL-MSCs *in vivo* has been determined in earlier studies. FLuc positive CL-MSCs were detected up to 10.9 ± 1.2 days in immunocompetent Balb/c mice (Martinez et al., 2013), 11 days after injection in xenogeneic murine and allogeneic human ELISPOT assays (Lilyanna et al., 2013), and 17.2 ± days for immunodeficiency SCID-beige mice (Martinez et al., 2013). It has thus been established that injected cells do not survive beyond 2–3 weeks, and do not form part of the new capillaries.

## Conclusion

CL-MSCs (US Patent number 9,737,568) implantation is safe and well-tolerated. The implantation of CL-MSCs demonstrates no toxicity associated morbidity and minimal local immune reaction to implantation. CL-MSCs have the capacity to stimulate angiogenesis in a rabbit hindlimb ischemia model. This early safety and efficacy data is encouraging and paves the way for future large animal studies or for clinical trials. The cGMP grade CL-MSCs or CorLiCyte has been approved by US-FDA for Phase-1 clinical trial for human study, bringing it a step closer to clinical application. These cells can potentially be used for patients with ischemic limbs in the near future.

## Data Availability Statement

The original contributions presented in the study are included in the article/supplementary materials, further inquiries can be directed to the corresponding author/s.

## Ethics Statement

The animal study was reviewed and approved by Institutional Review Board and SingHealth Institutional Animal Care and Use Committee, Singapore.

## Author Contributions

All authors contributed to the article and approved the submitted version.

## Conflict of Interest

TP is an inventor of Cord Lining Stem Cells technology, co-founder and major shareholder of CellResearch Corp. The remaining authors declare that the research was conducted in the absence of any commercial or financial relationships that could be construed as a potential conflict of interest.
